# Inhaled Ciclesonide for Community‐Based COVID‐19: A Placebo‐Controlled Randomised Trial

**DOI:** 10.1111/resp.70125

**Published:** 2025-10-07

**Authors:** Peter Wark, Ian C. Marschner, Owen Hutchings, Gemma Blunt, Greg J. Fox, Meg Jardine, Thomas Snelling, Arlen Wilcox, Emily Amico, Karen Allison, Kate Wilson, John Simes, Guy B. Marks

**Affiliations:** ^1^ School of Translational Medicine Monash University Melbourne Australia; ^2^ School of Medicine Health and Well Being University of Newcastle New Lambton Australia; ^3^ University of Sydney NHMRC Clinical Trials Centre Camperdown Australia; ^4^ Royal Prince Alfred Hospital Camperdown Australia; ^5^ School of Medicine University of New South Wales Liverpool Australia

**Keywords:** COVID‐19, inhaled corticosteroids, SARS‐CoV2

## Abstract

**Background and Objectives:**

Inhaled corticosteroids have been proposed as treatment for acute COVID‐19 infection in the community. We sought to determine the efficacy and safety of inhaled ciclesonide 320 mcg daily for 14 days compared to placebo in reducing the time to first recovery from all symptoms within 28 days of randomisation.

**Methods:**

We conducted a two‐arm, double blind, placebo‐controlled, community‐based randomised trial. Participants received inhaled ciclesonide 320 mcg daily or matching placebo for 14 days. The primary outcome was time to recovery of symptoms to day 28. An updated systematic review of all controlled trials of inhaled corticosteroids for acute COVID‐19 was then undertaken.

**Results:**

There were 189 people randomised and 185 completed treatment; 96% were COVID‐19 vaccinated. No difference was seen in the time to first recovery using a proportional hazards analysis, recovery rate ratio 1.04 (95% CI; 0.77, 1.40). The median time to recovery with ciclesonide was 7 days (95% CI 6–9), compared with placebo of 7 days (95% CI 6–9), *p* = 0.84. There were no significant differences in secondary outcomes, including time to sustained recovery and respiratory symptoms. The treatment was safe and well tolerated.

**Conclusion:**

In a highly vaccinated (> 90%) population exposed to Omicron variants of SARS‐CoV‐2, the addition of inhaled ciclesonide had no effect on accelerating recovery from acute symptoms. These trial results and updated combined evidence of placebo‐controlled trials do not support the use of inhaled corticosteroids for the treatment of COVID‐19.

**Trial Registration:**

ACTRN12620000566932

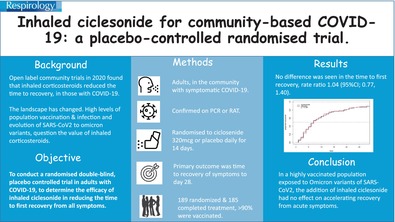

## Graphical abstract

We undertook a double‐blind, placebo‐controlled, randomised community trial of inhaled ciclesonide 320 mcg daily or placebo for 14 days. The primary outcome was time to recovery of symptoms to day 28. There were 189 people randomised and 185 completed treatment; 96% were COVID‐19 vaccinated. No difference was seen in the time to first recovery, 1.04 (95% CI; 0.77, 1.40). In a highly vaccinated (> 90%) population exposed to Omicron variants of SARS‐CoV‐2, the addition of inhaled ciclesonide had no effect on accelerating recovery from acute symptoms.

**See related** editorial

## 1 Introduction

While COVID‐19 has retreated from the headlines, it remains an important medical challenge for Australian practitioners. Antiviral therapy for COVID‐19 is an effective treatment option for the elderly and immunosuppressed, but presentations to primary care with acute viral respiratory symptoms remain one of the commonest reasons for urgent care, with people seeking reassurance and relief of symptoms [1]. Prior to the pandemic, Australian practitioners frequently prescribed short‐course inhaled corticosteroids with unclear efficacy in the context of acute respiratory infections [2].

SARS‐CoV2 emerged in 2019 and COVID‐19 rapidly developed into a global pandemic with extensive health and social consequences [3]. Early reports noted people with asthma were underrepresented in those hospitalised, and it was proposed that inhaled corticosteroids may be protective [4]. With no specific therapy available early in the pandemic, efforts were made to repurpose medicines, including inhaled corticosteroids. Open label trials conducted in 2020 in the UK found evidence that inhaled corticosteroids reduced the time to recovery, though they had an uncertain impact on hospitalisations or mortality [5, 6]. Omicron variants have predominated since mid‐2022 [7] and most Australians have now been infected, vaccinated, or both. Most Australians avoided infection in the early phases of the pandemic due to the implementation of effective public health interventions, but these were relaxed in January 2022 leading to an epidemic driven by Omicron variants in a population with a very high level of vaccination [8]. As a consequence, there is now greater uncertainty regarding the role of inhaled corticosteroids in the setting of early acute infection with SARS‐CoV2.

Our aim was to conduct a randomised double‐blind trial in adults with symptomatic, confirmed infection with SARS‐CoV‐2 to determine the efficacy and safety of inhaled ciclesonide 320 mcg daily for 14 days, compared to placebo, in reducing the time to first recovery from all symptoms within 28 days of randomisation.

## 2 Methods

### 2.1 Trial Design and Participants

This trial was conducted as an investigator‐initiated, two‐arm, double‐blind, placebo‐controlled, randomised trial, embedded within the BEAT COVID‐19 platform. The BEAT COVID‐19 platform trial was designed to be able to test multiple interventions in patients in the community with recently diagnosed COVID‐19 and allowing the use of different therapeutic care domains. This first trial domain aimed to assess the efficacy and safety of inhaled ciclesonide versus placebo.

Adults with confirmed SARS‐CoV‐2 infection, aged 18 years and older, who did not have clinical features of severe respiratory disease or require inpatient care at the time of enrolment, nor had specific contraindications to the use of inhaled corticosteroids, were eligible. Confirmation of SARS‐CoV‐2 required a positive PCR or rapid antigen test using a product approved by the Australian Therapeutic Goods Administration (TGA) and recruitment occurred as soon as possible following the commencement of symptoms or identification of infection (Table 1). Additional inclusion criteria were intended community management, the ability to use a metered dose inhaler after instruction, compliance with study procedures, and the ability to provide informed consent. Exclusion criteria included pregnancy or breastfeeding, and the current use of an inhaled corticosteroid‐containing preparation. Recruitment of people over 65 years of age and/or with a comorbid chronic illness such as cardiovascular disease or diabetes was prioritised.

**TABLE 1:** Subject characteristics.

	Ciclesonide (*n* = 95)	Placebo (*n* = 94)
Age (mean, SD)	57.6 (12.2)	56.8 (11.5)
Age < 65 years	62 (65.3%)	73 (77.7%)
Age ≥ 65 years	33 (34.7%)	21 (22.3%)
Sex (male, (*n*%))	37 (38.9)	34 (36.2%)
Vaccination		
Fully vaccinated^a^	93 (97.9%)	85 (90.4%)
Not fully vaccinated	2 (2.1%)	9 (9.6%)
Comorbidities present		
Coronary heart disease	13 (13.7%)	6 (6.4%)
Hypertension	30 (31.6%)	27 (28.7%)
Asthma	14 (14.7%)	10 (10.6%)
COPD	0	2 (2.1%)
Diabetes	14 (14.7%)	12 (12.8%)
Previous CVA	1 (1.1%)	1 (1.1%)
Active malignancy	1 (1.1%)	0
Immunosuppressive therapy	8 (8.4%)	9(9.6%)
Ethnicity		
Aboriginal/Torres Strait Islander	2 (2.1%)	4 (4.3%)
Caucasian	91 (95.8%)	85 (90.4%)
Maori/Pacifica	1 (1.1%)	0
East Asian	1 (1.1%)	2 (2.1%)
South Asian	0	1 (1.1%)
Other	0	2 (2.1%)
Symptom onset to randomisation		
Days, Mean, (SD)	2.9 (1.5)	3.0 (1.8)
>5 days	6 (6.4%)	9 (9.5%)
Presenting symptoms (self‐rated severity moderate or major)		
Cough	28 (29.5%)	28 (29.8%)
Fever	10 (10.5%)	10 (10.6%)
Fatigue	54 (56.8%)	51 (54.2%)
Breathlessness	8 (8.4%)	14 (14.9%)
Use of antiviral agents for SARS‐CoV2		
Molnupiravir	11 (11.6%)	10 (10.6%)
Nirmaltrevir/Ritonavir	9 (9.5%)	20 (21.2%)
Any antiviral	16 (16.8%)	22 (23.4%)

^a^ Vaccination status defined, fully vaccinated received two or more approved vaccines against SARS‐CoV2. Not fully vaccinated, less than 2 doses of an approved vaccine against SARS‐CoV2.

Randomisation was performed online via the study's clinical data system, Medidata. Permuted block randomisation was stratified by vaccination status (fully vs. not fully vaccinated) using a 1:1 ratio between ciclesonide and placebo. The primary analysis was by modified intention‐to‐treat, including all patients randomised to any trial treatment.

Recruitment occurred from two Australian sites, Royal Prince Alfred Virtual Hospital, Sydney and John Hunter Hospital Newcastle, New South Wales (NSW), with patients recruited and seen in the community remotely. At the time, all positive PCR results in NSW were reported to public health units, while the public was encouraged to upload the results of a positive rapid antigen test. Site staff contacted people who had been referred within 48 h to the public health unit with a positive result and within 5 days of symptom onset. Informed consent and eligibility were determined via telephone, with study documents sent by email. The interventional product and the placebo were identical in terms of appearance and taste. The interventional product and equipment were couriered to participants within 24 h of randomisation.

### 2.2 Study Assessments and Outcomes

Study assessments, training in the use of the device and equipment, and all visits occurred via videoconference. Participants were provided with access to a secure, online patient diary during the 28‐day study period. Participants were contacted by the research team daily in the first 14 days, then again on days 21, 28, 90 and 180 post‐randomisation. Where the online application or patient diary was not completed, the research team contacted the participant and/or their delegate to obtain the information. If the participant had been hospitalised, medical records were accessed to ascertain the required data to day 28. At baseline, participants completed a clinical assessment using equipment provided by the study team, including temperature, pulse rate, and oxygen saturation (SpO2) and completed a medical interview. From days 1 to 28, the participant diary recorded treatment adherence, COVID‐19 symptoms (cough, breathlessness, fatigue, fever), adverse events experienced, and return to normal function status (or recovery). On days 1 to 14 and day 28, each study participant completed a common cold questionnaire (CCQ). The CCQ provides 4 scores: general symptoms score, nasal symptoms score, throat symptoms score, and chest symptoms score [9]. EQ‐5D–5L quality of life questionnaire was collected on days 1 and 28. The EQ‐5D‐5L provides scores for five dimensions: mobility, self‐care, usual activities, pain/discomfort, and anxiety/depression. It also includes a self‐rated health vertical visual analogue scale [10, 11].

The primary outcome at the time of trial commencement was hospitalisation or death within 28 days of randomisation. This was changed to time to first recovery within 28 days in March 2023, when an interim blind data review found that there were no hospitalisations or deaths. Time to first recovery was defined as the number of days until the participant first reports an absence of all COVID‐19 symptoms (cough, breathlessness, fatigue, and fever) within 28 days after the date of randomisation. Secondary outcomes included: hospitalisation, death, early sustained recovery (absence of all symptoms on day 14 followed by no documented return of symptoms through day 28), time to sustained recovery, incidence of acute severe cardiorespiratory illness (SpO2 < 94% on room air, resting HR > 110 or systolic BP < 90 mmHg), the development of any one of these parameters becoming abnormal, pneumonitis, acute respiratory distress syndrome (ARDS), other COVID‐19‐related complications, duration of fever > 38°C, and all adverse events.

### 2.3 Systematic Review

We undertook a systematic review of all controlled trials of inhaled corticosteroids used for the treatment of acute COVID‐19 in order to put these trial results in context and to update the total randomised evidence in accordance with Lancet recommendations [12]. Results from the recently reported systematic review by Hsu et al. [13] were included, plus results from any other eligible trials (i.e., any randomised trial in patients with COVID‐19 comparing an inhaled steroid against a placebo or other comparator). Search terms used were COVID‐19 or SARS‐CoV2, and inhaled corticosteroids. We searched PubMed, Embase, the Cochrane Library, ClinicalTrials.gov, Scopus, Web of Science, and Google Scholar were searched to June 2024.

The systematic review used a random effects meta‐analysis with inverse variance weighting. Subgroup meta‐analysis was used to assess effect modification with study subgroups defined as placebo versus open label designs, and early versus later trial initiation. A test of interaction between study subgroup and treatment arm was used to test for significantly different treatment effects between study subgroups.

### 2.4 Statistical Analysis

The trial was set up as a platform trial with the aim of being able to assess multiple interventions for COVID‐19 treatment, and initially began as a randomised trial of inhaled ciclesonide versus placebo using a fixed sample size. A statistical analysis plan was finalised prior to any unblinded analysis. Power for assessing the primary objective was determined by the total number of participants that report an absence of symptoms within 28 days following randomisation. The primary analysis population was an intention‐to‐treat population including all participants in the treatment arm to which they were randomised, regardless of the actual treatment received.

The primary outcome was the time until the participant first reports an absence of all symptoms during the first 28 days following randomisation. To account for censoring due to some participants not reporting recovery by day 28, Kaplan–Meier curves were presented of the proportion of participants in each treatment group achieving recovery by each day until day 28. Median time to recovery in each group was presented, accompanied by a log‐rank test (stratified by vaccination status) to test for a difference between the treatment groups. The primary measure of treatment effect was the recovery rate ratio, which is the hazard ratio from a proportional hazards model adjusting for vaccination status. For binary endpoints (presence/absence), the treatment groups were compared using a chi‐squared test and odds ratio. Hazard ratio and odds ratio values greater than 1 favour the ciclesonide arm. Interim analyses were factored into the overarching platform trial design, with decision rules for superiority and futility. However, as there had been no hospitalisations or deaths when the primary endpoint was changed 6 months prior to the end of recruitment, there were no unblinded interim efficacy analyses conducted during the study.

In calculating the sample size, it was assumed that 80% of participants achieve recovery from all symptoms during the first 28 days following randomisation, so that 250 participants would provide a total of 200 events for the primary time‐to‐event analysis. Assuming a two‐sided alpha of 0.05, this number of events would provide at least 80% power for detecting a recovery rate ratio of 1.5 in favour of the ciclesonide. A fixed sample size of 250 (125 in each arm) was planned. As the participants were under close supervision with medication well tolerated, drop‐out was not expected to substantially impact these power calculations and was handled via censoring in the time‐to‐event analyses.

Prior to reaching the planned sample size, relevant external information became available when the ACTIV‐6 study was published, finding a lack of benefit for inhaled glucocorticoids [14]. On the basis of these results, the Trial Steering Committee made a decision in September 2023 to cease further recruitment. Treatment and follow‐up of all participants randomised prior to this decision was continued according to protocol.

During the trial, a member of the Steering Committee became eligible and participated in the domain. Neither they nor anyone involved in the conduct and analysis of the study was or is aware of their treatment allocation.

The study, an investigator‐initiated trial, was approved by the Sydney Local Health District Human Research Ethics Committee—Royal Prince Alfred Hospital Zone (2020/ETH01199) and the Royal Prince Alfred Hospital Research Ethics and Governance Office. The trial sponsor was the University of Sydney. Informed patient consent was obtained from study participants.

## 3 Results

**FIGURE 1:**
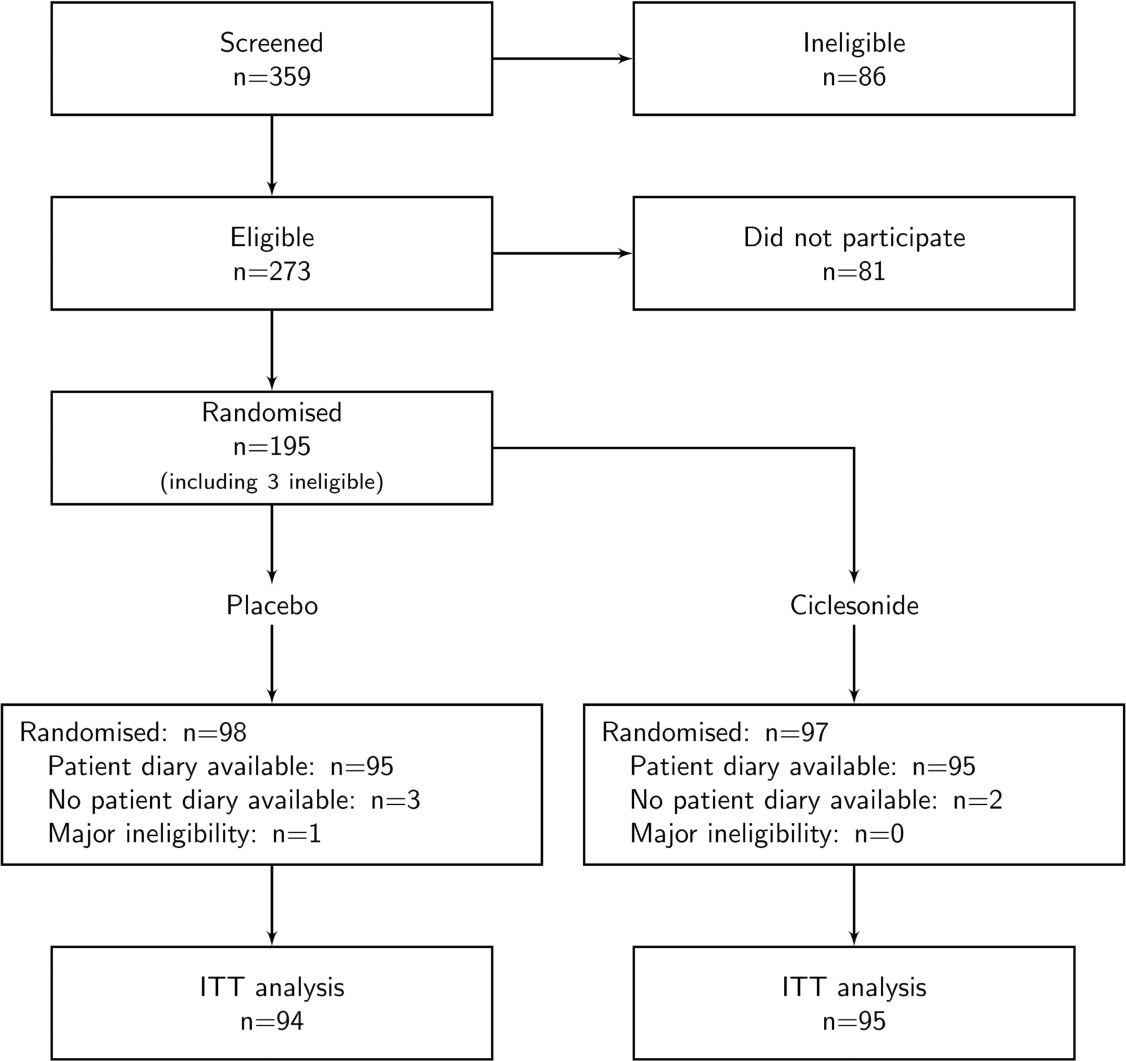
Flow chart for intention‐to‐treat analysis for all eligible participants.

There were 359 potential participants screened between 23 February 2022 and 19 September 2023, with 195 randomised, 98 to placebo, and 97 to ciclesonide. After exclusion of 1 major ineligibility and 5 participants with no patient diary available, there were 95 available with ciclesonide and 94 with placebo for the ITT analysis (Figure 1).

Participant characteristics are described in Table 1. Baseline symptoms, as anticipated, were mostly mild to moderate in severity, with no differences between the groups (Table 1). There were 38 (20%) participants also prescribed either molnupiravir or nirmatrelvir/ritonavir (23% assigned placebo and 17% assigned ciclesonide).

**FIGURE 2:**
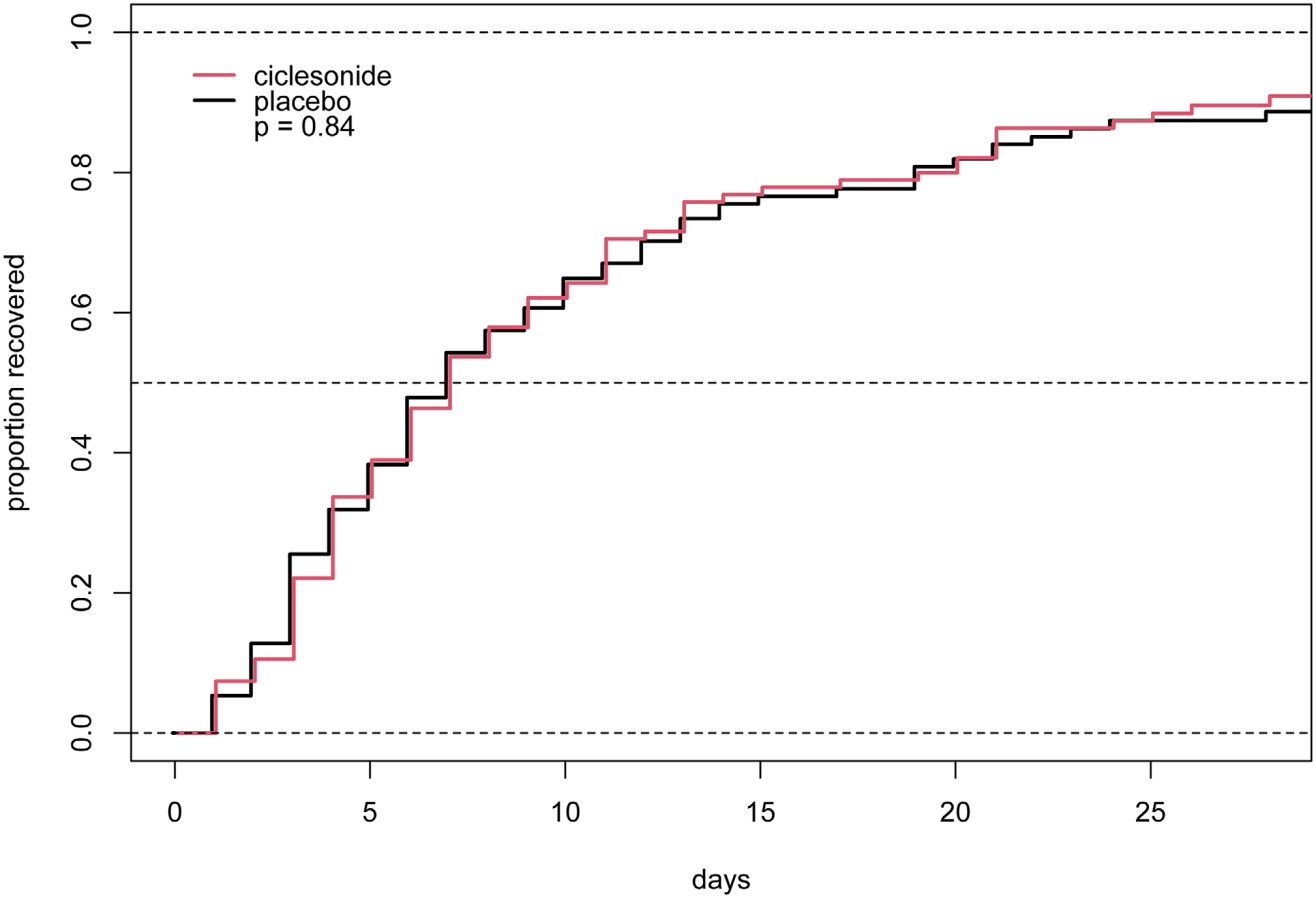
Time to first recovery. Kaplan‐Meier analysis, log‐rank test *p* = 0.84, values are the median time with 95% confidence intervals.

In terms of the primary outcome, the time to first recovery, there was no difference between those who received ciclesonide 86 (90.5%), compared to placebo 83 (88.3%), hazard ratio (HR) 1.04 (95% CI, 0.77, 1.4) (Figure 2). The median time to first recovery with ciclesonide was 7 days (95% CI; 6, 9) and with placebo was 7 days (95% CI; 6, 10). Predetermined subgroup analyses were performed based on vaccination status, age greater than or equal to 65 years, the presence of any comorbidity, COPD or asthma, those describing severe symptoms at presentation or the randomisation site, with no appreciable differences seen. Analysis per protocol also showed no differences in time to first recovery, HR 1.02 (95% CI; 0.75, 1.39).

There were no hospitalisations for COVID‐19, diagnosis of pneumonia or other acute cardiorespiratory illness. There was 1 death recorded in the placebo arm after an unrelated admission to a palliative care facility. In terms of early sustained recovery from symptoms, no differences were seen, odds ratio (OR) 0.78 (95% CI; 0.43, 1.4), median time to sustained recovery, ciclesonide 11 days (95%; 10, 15) and placebo 11 days (95% CI; 9, 14). There were no significant differences seen in time to sustained 3‐day recovery, HR 0.85 (95% CI; 0.63, 1.16), median on ciclesonide 12 days (95% CI 10, 15) and placebo 10 days (9, 13 days). By day 28 of the trial, most participants had achieved sustained recovery, 81/94 with placebo (86.2%) and 77/95 (81.1%) with ciclesonide, with no significant difference seen (HR 0.88, 95% CI; 0.65, 1.21). Symptoms experienced by the cohort were rated as mild to moderate for all categories, with the only severe symptoms reported being cough 11 (5.8%), fever 1 (0.01%), fatigue 28 (14.8%) and breathlessness 4 (2.1%), with no differences seen between the treatment groups (Table 2) (Table S1).

**TABLE 2:** Symptoms.

		Ciclesonide (*n* = 95)	Placebo (*n* = 94)	Comparison (odds ratio, 95% CI)
Cough	Mild or worse	90	85	0.52 (0.16, 1.58)
	Moderate or worse	42	41	0.98 (0.55, 1.74)
	Major	6	5	0.83 (0.23, 2.86)
Fever	Mild or worse	52	45	0.76 (0.43, 1.34)
	Moderate or worse	9	12	1.40 (0.56, 3.59)
	Major	0	1	NA
Fatigue	Mild or worse	89	86	0.72 (0.23, 2.17)
	Moderate or worse	59	54	0.82 (0.46, 1.47)
	Major	14	14	1.01 (0.45, 2.27)
Breathlessness	Mild or worse	58	60	1.13 (0.62, 2.03)
	Moderate or worse	13	15	1.20 (0.54, 2.71)
	Major	2	2	1.01 (0.12, 8.57)

*Note*: Odds ratio (OR) along with 95% confidence intervals are provided.

Self‐reported adherence to the interventions occurred with full compliance (13 or more days) reported by 80 with placebo and 84 with ciclesonide; partial compliance (1–12 days) was reported by 14 with placebo and 11 with ciclesonide. Adverse events were no different between the groups; they were reported as mild or moderate and did not result in treatment cessation or interruption (Table S1).

**FIGURE 3:**
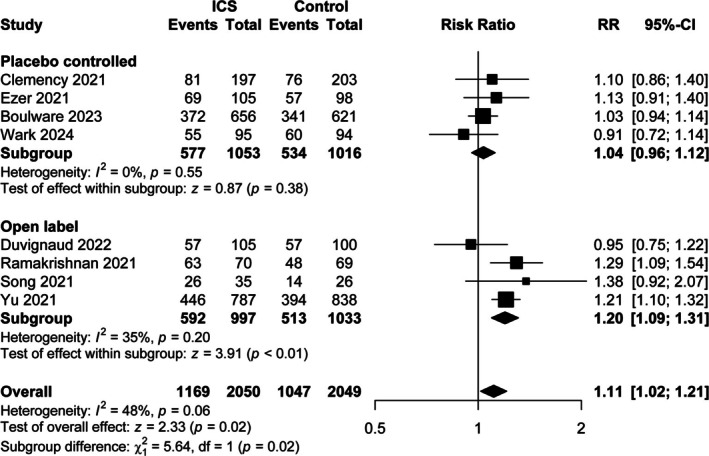
Metanalysis of controlled trials for inhaled corticosteroids in the treatment of acute COVID‐19. Summary data is provided for the Risk ratio (RR) along with 95% confidence intervals.

Our updated review of all controlled trials evaluating inhaled steroids in community‐based people with COVID‐19 revealed 12 original controlled trials and two meta‐analyses of these trials, which are detailed in Table 3. We performed a random effects meta‐analysis and subgroup meta‐analysis, dividing the trials into those that were open label and those that were placebo controlled (Figure 3). The combined analysis of all relevant trials suggests a symptom benefit of inhaled steroids with a risk ratio (RR) of 1.11 (1.05, 1.18). This was driven by the two large open label trials [5, 6], that also occurred early in the pandemic at a time before much COVID‐19 vaccination had occurred. When open label trials were compared with placebo controlled trials, there was evidence of a subgroup trial treatment interaction (*p* = 0.017) with an improved rate of symptom recovery among open label trials (RR 1.11 (95% CI; 1.05, 1.18)) but not among placebo controlled trials; RR 1.04 (95% CI; 0.96, 1.12) (Table S2). Based on the analysis of placebo controlled trials, there was 92.3% confidence that ciclesonide provides less than a 10% improvement in symptom recovery rate (RR < 1.1). Likewise, when trials that were undertaken in the early period of COVID‐19 [5, 6, 15, 16, 27, 28] were compared with our trial and ACTIV‐6 [14] (when vaccination rates were higher), similar evidence of a subgroup trial treatment interaction was seen (*p* = 0.013) with an improved rate of symptom recovery among the early trials, RR 1.17 (95% CI; 1.09, 1.26); but not among the later trials, RR 1.01 (95% CI; 0.93, 1.11) (Table S2).

**TABLE 3:** Randomised trials and meta‐analyses of inhaled corticosteroids for COVID‐19.

Study name	Placebo controlled	Treatment	Size	Result symptoms	Results events	Study period	Subject vaccination
Clemency [15]	Yes	Ciclesonide	400	±	++	June to Nov 2020	None
Duvignaud [16]	No	Ciclesonide	217	±		Dec 2020 to July 2023	None
Ezer [17]	Yes	Ciclesonide	203	±		Sept 2020 to June 2021	None
Song [18]	No	Ciclesonide	61	±		May 2020to March 2021	None
Terada‐Hirashima et al. [19]	No	Ciclesonide	89			April to September 2020	None
Brodin et al. [20]	No	Ciclesonide	98			June 2020 to May 2021	None
Suzuki et al. [21]	No (observational, propensity matched))	Ciclesonide	3638	NA	—	Jan to Sept 2020	None
Agusti et al. [22]	No	Budesonide	120			April 2020 to March 2021	None
Alsultan et al. [23]	No	Budesonide	35			August 2021	Assumed none, not stated
Ramakrishan et al. [5]	No	Budesonide	146	++	++	July to Dec 2020	None
Yu et al. [6]	No	Budesonide	1959	++	+	Nov 2020 to March 2021	None
Boulware et al. [24]	Yes	Fluticasone	1277	±	−	August 2021 to Feb 2022	65% reporting 1 or more vaccine doses
**Wark**	**Yes**	**Ciclesonide**	**195**	±	±	**2022–3**	> 95% ×2 vaccine doses
Hsu et al. [25]	Meta‐analysis			++	+	Search up to March 2023	NA
Badi et al. [26]	Meta‐analysis			+	+	Search up to Aug 2022	NA

*Note*: Symptoms—recovery within 7 and within 14 days. Events—reduced urgent care, hospitalisation, or death. ++ significant benefit; + trend in favour; ± neutral; − trend against. Bold values represent the study described in this manuscript.

## 4 Discussion

In a community‐based study of adults with confirmed infection with SARS‐CoV‐2, treatment with inhaled ciclesonide did not result in a faster time to recovery from acute infection compared to placebo. There were no significant differences observed in terms of the time to recovery or in any of the secondary outcomes. Most participants experienced only mild to moderately severe symptoms, and only one participant was admitted to a health care facility as a consequence of infection; they had a pre‐existing terminal condition and were treated palliatively. Over 80% of participants achieved sustained resolution of their symptoms by day 28, while only 23% of participants reported symptoms that they regarded as major during the time of the study. Inhaled ciclesonide appeared safe and well tolerated.

Previous experience from the 2009 influenza pandemic identified people with asthma as being at heightened risk of hospitalisations, especially those not using inhaled corticosteroids [29, 30]. Virus infections are a common trigger for acute exacerbations in people with asthma, though this risk is reduced with the use of inhaled corticosteroids [14]. It therefore came as a surprise early in the pandemic that people with asthma were underrepresented among those hospitalised with COVID‐19, with varying theories proposed, including the potential protective effect of inhaled corticosteroids [31]. This resulted in an open label randomised trial of inhaled budesonide in early 2020, targeting people in the UK, in the community, at the early stage of infection, with the aim of reducing severe respiratory illness. In this context, the trial did demonstrate a reduction in acute deterioration requiring hospitalisation and a shorter course of illness with budesonide [5]. This led to the larger “Principle trial” that was also not placebo‐controlled and similarly demonstrated improved time to recovery, though not hospitalisation [6]. As a result, inhaled corticosteroids became accepted in many jurisdictions as a treatment option early in COVID‐19. Subsequently, the pandemic evolved quickly, with SARS‐CoV‐2 Omicron variants dominating from mid‐2022 [7] along with large proportions of the population becoming vaccinated or with some immunity as a result of previous infection.

A series of randomised trials of inhaled corticosteroids has now been undertaken for community‐based people with COVID‐19. A meta‐analysis of 11 trials shows inconsistent findings, though suggesting there was still a benefit in symptomatic relief, though not the need for hospitalisation [13]. The most recent trial of inhaled fluticasone did not show any benefit in symptom improvement [24]. This trial was similar to our own, recruiting a slightly older population in the US but only relying upon a clinical diagnosis for enrolment. Their primary outcome, the time to sustained recovery, defined as the third of 3 consecutive days without symptoms, with hospitalisation or death by day 28, did not show any benefit of inhaled steroids [24].

Our trial results did not show a benefit of inhaled steroids, but with wide confidence intervals on the primary outcome of the rate of recovery from symptoms, a clinically important benefit is not excluded. We therefore undertook an updated meta‐analysis of all randomised controlled trials using inhaled corticosteroids, including our own results in accordance with the Lancet recommendation of putting trial results in context [12]. Initial trials captured earlier SARS‐CoV2 variants and populations largely naïve to infection. They also were often open labelled. However, blinding is an important tool to reduce bias. As the primary outcomes assessed were symptom‐based, the risk of bias with unblinded therapy would have to be regarded as high. These combined results showed evidence of heterogeneity of treatment effect when trials were grouped according to open labelled versus placebo controlled, and with no evidence of treatment benefit when restricted just to the placebo controlled trials. Similarly, significant heterogeneity was found when trials were grouped by those occurring early versus later in the pandemic. No evidence of treatment benefit was observed among the more recent trials (at a time when vaccination rates were high) (Table 3 and Figure 3). It needs to be acknowledged that all open labelled trials were conducted earlier in the pandemic, making it hard to deduce which of these effects (placebo control or evolution of the virus) is responsible for the lack of benefit of inhaled steroids. Nonetheless, lack of blinding is a recognised risk of bias in systematic reviews and therefore may be the more plausible explanation.

Inhaled corticosteroids are frequently prescribed in the context of an acute respiratory illness [2]. This is most often in response to the presence of symptoms such as cough. A systematic review identified four randomised trials and found there was inconclusive evidence that they reduced either acute or persistent cough, though it did find they appeared safe [32]. Cough was a frequent symptom that we also recorded, but even in those who rated it as moderate and severe, inhaled ciclesonide had no effect. We did not assess the risk of chronic cough. One cohort study of 104 people assessed with persistent cough present for 8 weeks following COVID‐19 infection (Omicron) found those who had been treated with inhaled corticosteroids were not less likely to experience cough, though treatment with inhaled corticosteroids was not randomised and there was a high proportion of participants with bronchial hyperresponsiveness [27]. One Japanese study reported persistent cough at 3 months in 8.8% of people who had been admitted with COVID‐19 [28]. The prevalence of chronic cough following mild acute disease is uncertain, and the role of inhaled corticosteroids in this context remains unclear.

Our trial has several strengths as well as limitations. Conducting intervention trials for acute viral respiratory illnesses is challenging, with the need for people to be identified as soon as they become unwell and the problem of confirming infection being present. At the time of recruitment, people in NSW had heightened motivation to undergo either PCR testing or self‐administer a rapid antigen test, both of which were reportable, leading to a very high rate of reporting. This allowed researchers to identify and approach people with a high level of certainty that they had acute COVID‐19. The trial was wholly conducted virtually, including consent, the distribution of the investigational product and all equipment, and the recording of symptoms, along with support from research staff; this led to a high rate of recruitment and adherence to study processes. The participants and staff were also blinded to the intervention, giving us a high level of certainty in the quality of the data. The trial set up here provided a practical approach to community‐based illness via telehealth that could be considered in future intervention trials for acute respiratory infections.

Conversely, the trial had several limitations. Challenges in first setting up the trial and reaching agreement for remote access delayed the start and pace of recruitment, with a smaller sample size than planned. This meant moderate but clinically important benefits of treatment could not be excluded. Nevertheless, when put in the context of all relevant quality‐controlled trials in the setting of vaccinated populations of with more recent variants of the virus, the updated results and analyses provide clear evidence to guide future care.

In conclusion, in an adult vaccinated population with confirmed infection with SARS‐CoV‐2, Omicron variants, the addition of inhaled ciclesonide had no significant benefit in the time to first recovery or any other improvement in acute symptoms. Therefore, we do not recommend the use of inhaled corticosteroids in the current treatment of acute COVID‐19. These results provide clarity for Australian practitioners to reconsider prescribing inhaled corticosteroids for acute COVID‐19.

## Author Contributions

**Peter Wark:** conceptualization (equal), investigation (equal), methodology (equal), project administration (equal), supervision (equal), writing – original draft (equal), writing – review and editing (equal). **Ian C. Marschner:** data curation (equal), formal analysis (equal), methodology (equal), project administration (equal). **Owen Hutchings:** investigation (equal), supervision (equal). **Gemma Blunt:** data curation (equal), methodology (equal). **Greg J. Fox:** investigation (equal), methodology (equal), supervision (equal). **Meg Jardine:** investigation (equal), methodology (equal), supervision (equal), writing – review and editing (equal). **Thomas Snelling:** investigation (equal), methodology (equal), writing – review and editing (equal). **Arlen Wilcox:** investigation (equal), methodology (equal), writing – review and editing (equal). **Emily Amico:** methodology (equal), project administration (equal). **Karen Allison:** investigation (equal), methodology (equal), writing – review and editing (equal). **Kate Wilson:** investigation (equal), methodology (equal), writing – review and editing (equal). **Guy B. Marks:** conceptualization (lead), formal analysis (equal), funding acquisition (lead), methodology (equal), project administration (lead), resources (equal), supervision (lead), writing – review and editing (equal). **John Simes:** conceptualization (equal), formal analysis (equal), funding acquisition (equal), investigation (equal), methodology (equal), project administration (equal), writing – original draft (equal), writing – review and editing (equal).

## Ethics Statement

This clinical trial was prospectively registered with the Australian Clinical Trials Registry; **Trial Registration** ACTRN12620000566932. The study, an investigator‐initiated trial, was approved by the Sydney Local Health District Human Research Ethics Committee—Royal Prince Alfred Hospital Zone (2020/ETH01199) and the Royal Prince Alfred Hospital Research Ethics and Governance Office. The trial sponsor was the University of Sydney.

## Conflicts of Interest

The interventional product and the placebo were supplied free of cost by Chiesi pharmaceuticals.

## Supporting information


**Data S1:** Tables Information.

## Data Availability

The authors are prepared to share deidentified data upon request to the corresponding author.
