# Supplementary material for: Inhaled Ciclesonide for Community‐Based COVID‐19: A Placebo‐Controlled Randomised Trial
Source: Respirology. 2025 Oct 7;31(1):82–90. doi: 10.1111/resp.70125 (PMC12783969; doi:10.1111/resp.70125)
Supplement: Supplementary file 1 — Data S1: Tables Information. [file RESP-31-82-s001.docx]

## Supplementary Table S1

|  | Placebo | Ciclesonide |
| --- | --- | --- |
| Seizure | 0 | 1 |
| Flu like illness | 1 | 2 |
| Headache | 1 |  |
| Respiratory | 2 | 1 |
| Rash | 2 |  |
| Mucositis/oral irritation | 0 | 1 |
| Vertigo | 0 | 1 |
| Sinusitis/ nasal symptoms | 0 | 1 |

**Legend;** total numbers.

## Supplementary Table S2.

Random effects subgroup meta-analysis of the effect of inhaled corticosteriods (ICS) on day 14 recovery, comparing placebo controlled and open label studies.

| Subgroup of Trials | Risk Ratio (95% CI) | Interaction p-value |
| --- | --- | --- |
| **Trial Design** |  | **0.017** |
| Open Label Trials | 1.11 (1.05, 1.18) |  |
| Placebo Controlled Trials | 1.04 (0.96, 1.12) |  |
| **Trial Period*** |  | **0.013** |
| Early Trials | 1.17 (1.09, 1.26) |  |
| Later Trials | - 1. (0.93, 1.11) |  |

**Legend;** * Early trials – commenced before Aug 2021; Later trials – commenced after August 20
